# Association of mitochondrial homeostasis and dynamic balance with malignant biological behaviors of gastrointestinal cancer

**DOI:** 10.1186/s12967-023-03878-1

**Published:** 2023-01-16

**Authors:** Ao-ran Liu, Zhi Lv, Zi-wei Yan, Xiao-yang Wu, Li-rong Yan, Li-ping Sun, Yuan Yuan, Qian Xu

**Affiliations:** 1 Tumor Etiology and Screening Department of Cancer Institute and General Surgery, The First Hospital of China Medical University, No. 155 North NanjingBei Street, Heping District, Shenyang, 110001 Liaoning People’s Republic of China; 2 Key Laboratory of Cancer Etiology and Prevention in Liaoning Education Department, The First Hospital of China Medical University, Shenyang, 110001 China; 3 Key Laboratory of GI Cancer Etiology and Prevention in Liaoning Province, The First Hospital of China Medical University, Shenyang, 110001 China

**Keywords:** Mitochondria, Mitochondrial dynamic, Mitochondrial homeostasis, Gastrointestinal cancer, Fission, Fusion, Mitophagy, Mitochondrial quality control, Mitochondrial biogenesis

## Abstract

Mitochondria determine the physiological status of most eukaryotes. Mitochondrial dynamics plays an important role in maintaining mitochondrial homeostasis, and the disorder in mitochondrial dynamics could affect cellular energy metabolism leading to tumorigenesis. In recent years, disrupted mitochondrial dynamics has been found to influence the biological behaviors of gastrointestinal cancer with the potential to be a novel target for its individualized therapy. This review systematically introduced the role of mitochondrial dynamics in maintaining mitochondrial homeostasis, and further elaborated the effects of disrupted mitochondrial dynamics on the cellular biological behaviors of gastrointestinal cancer as well as its association with cancer progression. We aim to provide clues for elucidating the etiology and pathogenesis of gastrointestinal cancer from the perspective of mitochondrial homeostasis and disorder.

## Introduction

Latest research indicates that gastrointestinal cancer is a malignant tumor of digestive system with the highest morbidity and mortality worldwide. Among them, gastric cancer has the fifth incidence and the fourth mortality rate. Colorectal cancer is a malignancy with the third incidence and the second mortality rate [1]. Exploration for the etiology and pathogenesis of gastrointestinal cancer would improve the individualized therapy and survival of cancer patients with great scientific significance and application value.

In recent years, a novel molecular perspective for studying tumorigenesis is the communication between cell organs and nucleus as well as the roles of cell organs in maintaining homeostasis [2]. Mitochondrion is a semi-autonomous organ in cells with its own DNA, making it a special organ co-regulated by nuclear DNA and mtDNA [3]. Mitochondria are organelles with double-layered membranes in eukaryotic cells taking the main place for cellular aerobic respiration. They are not only involved in energy metabolism but also generate reactive oxygen species (ROS) through electron transport controlling cell apoptosis and other functions [4, 5]. Mitochondria continuously fissure and fuse forming a homeostasis called mitochondrial dynamics [6–8]. As a highly dynamic organelle, mitochondrion maintains homeostasis by the aid of mitochondrial quality control (MQC) system. MQC system is in composed of mitochondrial dynamic balance (fusion and fission), biogenesis and mitophagy [9]. Mitochondrial fusion and fission can not only regulate mitochondria independently but also interact with other balances in MQC, forming a regulatory network to synergistically keep the normal function of mitochondria. When the dynamics is disrupted, MQC becomes abnormal and the homeostasis will be damaged resulting in structural damage, mitochondrial dysfunction and eventually tumorigenesis. It has been paid increased attention that mitochondrial dynamics functions in cancer genesis and progression.

This review briefly introduced the normal and aberrant status of mitochondrial dynamics, sorted out the role of mitochondrial dynamics in maintaining mitochondrial homeostasis and elaborated the effects of mitochondrial dysfunction on cellular biological behaviors and progression of gastrointestinal tumor. We aim to provide theoretical basis for elucidating the etiology and pathogenesis of gastrointestinal tumor from the perspective of mitochondrial homeostasis and disorder.

## Roles of mitochondrial dynamics in maintaining mitochondrial homeostasis

### Basis of mitochondrial homeostasis maintenance–mitochondrial quality control

Mitochondrial quality control (MQC) system acts as a “monitoring checker” and monitors dynamic balance to meet the energy demands and metabolic activity of cells maintaining mitochondrial homeostasis [10]. Mitochondria manage the process of fission and fusion, biogenesis and mitophagy through MQC to eliminate damaged or aging mitochondria and synthesize new mitochondria, ensuring the stability of mitochondrial quantity, morphology, quality and internal environment.

Substantial evidence suggested that disrupted mitochondrial homeostasis played a critical role in cancer genesis and progression. Across all the four parts of MQC including fusion, fission, biogenesis and mitophagy, disorder appears in any one of them that cannot be corrected could damage the homeostasis causing diseases related to mitochondrial dysfunction. The expression levels of relevant molecules are associated with disease progression. Detection for them has the potential to be diagnostic biomarkers and therapeutic targets of diseases.

#### Balance of mitochondrial dynamics (fusion and fission)

Mitochondrial dynamics keeps the balance and remodels mitochondrial network by fusion and fission to adapt to the needs of various cells and tissue, which is essential for the regulation of mitochondrial homeostasis.

**Fig. 1:**
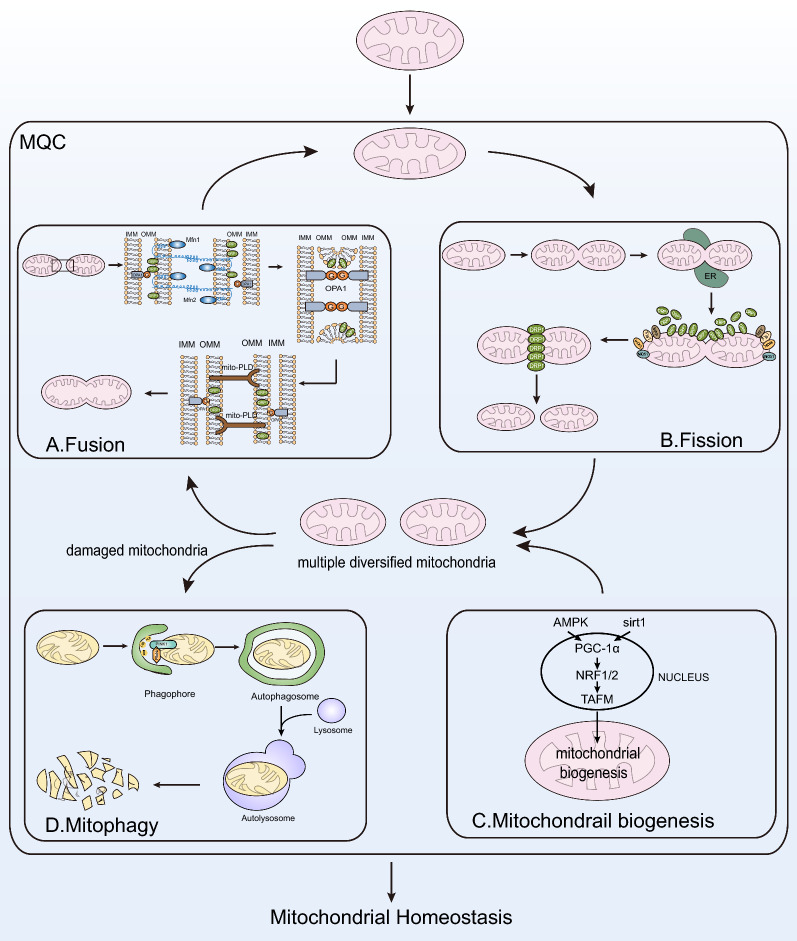
Mitochondrial homeostasis is maintained by MQC system. Mitochondrial quality control system is composed of the balance between fusion (**A**) and fission (**B**), and between biogenesis (**C**) and mitophagy (**D**). **A**. Fusion. Mitochondria mediate fusion of IMM and OMM through OPA1 and MFN1/2. **B**. Fission. Mitochondria complete fission through fission-related factors such as DRP1 and dynein. **C**. Mitochondrial biogenesis. AMPK and SIRT1 pathways activate the PGC-1α/NRF1/2/TAFM axis and affect mitochondrial biogenesis. **D**. Mitophagy. Mitochondrial mitophagy is initiated to eliminate damaged mitochondria generated from disrupted fission and fusion

Fusion enables damaged and healthy mitochondrial contents to fully mix making metabolites exchanged. That may help to reduce the stress, prevent excessive fragment and maintain the morphology of mitochondria. Mitochondrial expansion in a network strengthens the oxidation capacity of metabolically active cells to increase ATP production catering to the high energy demands of cells [11, 12]. Mitochondrial fusion requires outer membrane (OMM) fusion mediated by mitofusion 1/2 (MFN1/2) and inner membrane (IMM) fusion mediated by optic atrophy 1 (OPA1), a GTPase. During OMM fusion, MFN1/2 can impel two close mitochondria to combine together by their interaction. Then mitochondrial phospholipase D (mitoPLD) alters the composition of membranes, forms smaller lipids as the second messenger to activate signaling pathways and hydrolyze allostery, and enables GTPases to mediate the fusion of mitochondrial membranes [13, 14]. In IMM fusion, OPA1 acts on mitochondrial IMM to affect its stability [15]. After OMM and IMM fusion, damaged mitochondria are replaced with newborn mitochondria to buffer the internal pressure (Fig. 1A).

Mitochondrial fission also plays an indispensable role in maintaining mitochondrial morphology, which is a multi-step process coordinated by multiple factors [16]. DRP1 (dynamin-related protein 1) in cytoplasm is recruited to OMM, the self-assembled spherical oligomers slowly wrap the mitochondria and cut off the mitochondria to complete the fission. It requires the interaction among DRP1, mitochondrial adaptor proteins including mitochondrial fission factor (MFF), mitochondrial fission protein1 (FIS1), mitochondrial dynamic protein of 49kD (MiD49) and mitochondrial dynamic protein of 51kD (MiD51) as well as the organelle endoplasmic reticulum. First, mitochondria associate with endoplasmic reticulum (ER) to communicate information. Second, ER mediates DRP1 to bind to mitochondrial adaptor protein on OMM making DRP1 assembled into a spiral at the fission site. Finally, DRP1 hydrolyzes GTP to divide a mitochondrion into two sub-mitochondria (Fig. 1B).

Fusion and fission in mitochondria convert into each other under constant movement to form dynamic balance, which coordinately regulates mitochondrial morphology and enables mitochondria to respond accordingly to the changes of intracellular environment [17]. On the one hand, when cells are metabolically active with higher metabolic demands, mitochondria are elongated by fusion, the area of mitochondrial cristae increases and more ATP is generated for required energy [18]. On the other hand, aging cells produce excessive ROS and damaged mitochondria accumulate continuously. Mitochondrial fission is initiated to fragment damaged mitochondria into smaller pieces facilitating the clearance by mitophagy to prevent further damage of ROS [19]. The subtle regulation between fusion and fission ensures mitochondrial adaptability and keeps mitochondrial contents in a dynamic balance.

#### Balance of mitochondrial biogenesis and mitophagy

Mitochondrial biogenesis and mitophagy are suggested to be opposite processes constructing mitochondrial turnover together [20]. In mitochondrial biogenesis, proteins originated from nucleus enter into mitochondria to promote mitochondrial newborn and increase their number. While in mitophagy, damaged or aging mitochondria are self-selectively removed or degraded. Relative balance between the two courses maintains number stability and metabolic homeostasis in the mitochondrial pool [21].

Mitochondrial biogenesis supplies “fresh blood” to the mitochondrial pool and guarantees mitochondrial activity [22]. PGC-1α (PPARγ co-activator-1alpha)/NRF (nuclear respiratory factor)/TFAM (mitochondrial transcription factor A) is currently recognized as the key regulatory axis of biogenesis. AMPK-PGC-1α and SIRT1 (sirtuin1)-PGC-1α are the two major pathways regulating mitochondrial biogenesis [22]. In the AMPK-PGC-1α axis, AMPK can be activated along with increased AMP resulting in PGC-1α phosphorylation and the activation of whole pathway [23]. As for the SIRT1 (sirtuin1)-PGC-1α axis, SIRT1 (sirtuin1) is activated due to increased NAD/NADH and PGC-1α is further activated by SIRT1 deacetylation [24]. PGC-1α stimulated by AMPK and SIRT1 binds to NRF1/2 up-regulating TFAM. The elevation of transcription factor TFAM promotes mtDNA replication and transcription. Mounting mtDNA cooperates with mitochondrial protein encoded by nDNA (nucleus DNA) to trigger mitochondrial biogenesis [25] (Fig. 1C).

During mitochondrial mitophagy, damaged mitochondria are specifically and selectively degraded by autophagy in cells, which is a self-protective process [26, 27]. PINK1 (PTEN-induced putative kinase protein1) and PARK2 (cytosolic ubiquitin E3 ligase, Parkin) are the key proteins in the course. Mitophagy initiated by them could protect mitochondria from oxidative stress, prevent ROS overproduction and complete self-renewal in metabolically active tissue such as BAT (brown adipose tissue) [28] (Fig. 1D).

The balance between mitochondrial biogenesis and mitophagy is necessary for cells to recover from stressful and damaged status [29]. In mitochondrial biogenesis, PGC-1α and NRF1 were shown to up-regulate protein expression of mitophagy receptor FUNDC1, while knockout of FUNDC1 caused PGC-1α down-regulation. Therefore, mitochondrial biogenesis was accompanied with mitophagy and abnormal mitophagy could inhibit biogenesis as feedback. In some cells with activated mitophagy, the activation of biogenesis was also discovered [28, 30, 31]. These may illustrate that mitochondria require biogenesis to adapt to cellular changes in energy demands caused by mitophagy, which is regarded as an anabolic-catabolic balance [32].

### Central roles of mitochondrial dynamics in mitochondrial homeostasis

Mitochondrial dynamics structured by fusion and fission has been found to have a certain regulatory effect on mitochondrial biogenesis and mitophagy, which constitute the core of mitochondrial homeostasis.

Numerous studies have shown that mitochondrial fusion and fission are closely associated with mitophagy. Disrupted mitochondrial dynamics affects the degradation of damaged organelles by mitophagy. When mitochondria undergo asymmetric fission, DRP1 segregates components of damaged mitochondria into a depolarized sub-organelle for mitophagy [33]. Hence, mitophagy is initiated immediately after mitochondrial fission [34]. Although DRP1 was suggested to be necessary for mitophagy [35], some studies demonstrated that mitophagy might be independent from it [36, 37]. Jonathan et al. proposed a new point that fission did not promote mitophagy directly but protect healthy mitochondrial subdomains from unexamined PINK1-Parkin feedback [38]. And mitochondrial fragmentation due to the loss of fusion resisted the turnover of mitophagy. MFN2 is an important regulator of the PINK-MFN2-Parkin mitophagy axis [39]. It drives mitophagy through ubiquitination by PINK1 and Parkin [40]. Therefore, the imbalance of fission and fusion can result in significant changes in mitochondrial mitophagy.

The importance of mitochondrial dynamics for biogenesis has also been emphasized by plenty of research [41, 42]. The reduction of DRP1 and FIS1 by an inhibitor of mitochondrial fission (Mdivi-1) increased the key regulators of biogenesis including PGC-1α, NRF1, NRF2 and TFAM [43]. A latest study showed that the activation of mitochondrial fission downregulated PGC-1α/PPARα signaling in hepatocellular carcinoma (HCC) cells and inhibited SIRT1 driving metabolic reprogramming in HCC [44]. Alteration in mitochondrial biogenesis can affect the dynamics in turn? In cardiomyocytes, the elevated expression of NRF2 could down-regulate DRP1 and up-regulate MFN2 leading to excessive mitochondrial fusion [45]. Ding et al. found that PGC-1α could bind to the transcriptional promoter of DRP1 to inhibit DRP1-mediated fission relieving diabetes-induced cardiac insufficiency [46]. Natia et al. also reported that PGC-1α inhibited DRP1 expression and improved myocardial ischemia–reperfusion injury [47]. In addition, PGC-1α inhibited mitophagy by attenuating MFN2 ubiquitination and degradation [48]. All these suggested that mitochondrial dynamic balance was closely associated with biogenesis making impacts on mitochondrial homeostasis.

### Mitochondrial dysfunction and cancer based on big data analysis

Mitochondrial dysfunction is closely associated with tumorigenesis. It has been proven to affect oncogenic pathways and multiple cancer phenotypes (Table 1).

**Table 1:** The causes and consequences of mitochondrial dysfunction in different cancer

Gene	Year	Cancer type	Causes of mitochondrial dysfunction	Consequences	References
DRP1	2022	Pituitary adenomas	Upregulation	Inhibit the tumor growth	[60]
DRP1	2022	Esophageal squamous cell carcinoma	Upregulation	Trigger autophagy and promote ESCC progression	[61]
DRP1	2022	Hepatocellular carcinoma	Upregulation	Promote tumor metastasis	[62]
DRP1	2022	Colon cancer	Upregulation	Promote fatty acids-induced metabolic reprograming	[63]
DRP1	2022	Head and neck cancer	Upregulation	Promote tumor growth and metastasis and induce glycolysis	[64]
DRP1	2021	Pancreatic cancer	Upregulation	Maintain stemness-related features, such as self-renewal, tumorigenicity, and invasiveness	[65]
DRP1	2021	Colon cancer	Upregulation	Promote colon tumorigenesis	[66]
DRP1	2021	Cancer stem cells	Upregulation	Promote stemness and chemoresistance	[67]
DRP1	2021	Colorectal cancer	Upregulation	Promote tumor progression and metabolic reprogramming	[68]
DRP1	2021	Hepatocellular carcinoma	Downregulation	Inhibit the proliferation and migration	[69]
DRP1	2020	Lung cancer	Upregulation	Contribute to baicalein-induced apoptosis and autophagy	[70]
DRP1	2020	Prostate cancer	Upregulation	Regulate mitochondrial metabolic reprogramming	[71]
DRP1	2020	Ovarian cancer	Upregulation	Promote pancreatic cancer growth and metastasis	[72]
DRP1	2020	Pancreatic cancer	Upregulation	Promote pancreatic cancer growth and metastasis	[73]
DRP1	2020	Hepatocellular carcinoma	Downregulation	Promote mitophagy	[74]
DRP1	2020	Uterine	Upregulation	Promote metastasis	[75]
DRP1	2020	Ovarian cancer; colorectal cancer	Upregulation	Promote proliferation	[76]
DRP1	2020	Lung cancer	Upregulation	Promote proliferation and metabolic reprogramming	[77]
DRP1	2020	Renal cell carcinomas	Upregulation	Promote migration and invasion	[78]
DRP1	2020	Breast cancer	Upregulation	Positively correlate with infiltration levels of immune system	[79]
DRP1	2018	Breast cancer	Upregulation	Contribute to IR-783-induced apoptosis	[80]
FIS1	2022	Gastric adenocarcinoma	Upregulation	Promote metastasis	[81]
FIS1	2021	Hepatocellular carcinoma	Phosphorylation	Promote metastasis	[81]
FIS1	2018	Esophageal cancer	Downregulation	Decrease the mitochondrial membrane potential	[82]
MFN1	2022	Hepatocellular carcinoma	Downregulation	Inhibit reprogramming cellular metabolism	[83]
MFN1	2022	Liver cancer	Downregulation	Promote aerobic glycolysis and proliferation	[84]
MFN1	2020	Hepatocellular carcinoma	Downregulation	Promote cell proliferation, invasion and migration capacity by modulating metabolic reprogramming	[85]
MFN2	2022	Ovarian cancer	Upregulation	Promote autophagy, reduce ROS, and suppress OC progression	[86]
MFN2	2021	Thyroid cancer	Upregulation	Inhibit cell migration and invasion	[87]
MFN2	2021	Pancreatic cancer	Upregulation	Inhibit cell growth while promoting the formation of apoptotic bodies	[88]
MFN2	2021	Cervical cancer	Downregulation	Reduce the proliferation, colony formation ability, migration, and in vivo tumorigenesis	[89]
MFN2	2018	Pancreatic cancer	Downregulation	Induce apoptosis, metabolism disorder and migration impairment	[90]
MFN2	2021	Lung cancer	Phosphorylation	promote cell proliferation	[91]
NRF1	2021	Renal cell carcinoma	Upregulation	Promote cell proliferation	[92]
NRF1	2020	Liver cancer	Downregulation	Promote proliferation, invasion and metastasis	[93]
NRF1	2018	Insulinoma	Downregulation	Acquire aggressiveness and chemoresistance	[94]
NRF2	2021	Head and neck cancer	Upregulation	Promote proliferation and metabolic reprogramming	[95]
OPA1	2022	Breast cancer	Downregulation	Reduce proliferation, migration, and invasion	[53]
OPA1	2022	Pancreatic ductal adenocarcinoma	Upregulation	Acquire stemness capacity	[96]
OPA1	2020	Liver cancer	Upregulation	Alter metabolism and promote tumor cell growth	[54]
PGC-1α	2021	Pancreatic cancer	Upregulation	Promote metabolic reprogramming and stemness features	[97]
PGC-1α	2020	Melanoma	Upregulation	Promote metastasis	[98]
PGC-1α	2021	Hepatocellular carcinoma	Upregulation	Inhibit cell proliferation and induce apoptosis	[99]
PGC-1α	2021	Pancreatic cancer	Upregulation	Promote growth and invasion	[100]
PGC-1α	2020	Breast cancer	Upregulation	Inhibit glycolytic metabolism and proliferation	[101]
PINK1	2021	Breast cancer	Upregulation	Trigger PINK1/Parkin-mediated mitophagy and induce mitochondrial apoptosis	[102]
PINK1	2021	Colon tumor	Upregulation	Suppress colon tumor growth	[103]
PINK1	2021	Bladder cancer	Downregulation	Promote bladder tumor cell growth	[104]
PINK1	2019	Lung cancer	Downregulation	Reduce cancer cell proliferation, increase cell death, reduce ATP production, inhibit mitophagy	[105]
SIRT1	2022	Ovarian cancer	Upregulation	Increase mitochondrial activity	[106]
SIRT1	2019	Prostate cancer	Upregulation	Promote metastasis	[107]
SIRT1	2019	Liver cancer	Upregulation	Enhance the metabolic flexibility of liver cancer stem cells	[108]
SIRT1	2019	Rectal cancer	Upregulation	Inhibit growth	[109]
TFAM	2022	Lung cancer	Downregulation	Inhibit growth	[110]
TFAM	2021	Colorectal cancer	Upregulation	Promote the proliferation	[111]
TFAM	2021	Head and neck tumorigenesis	Downregulation	Enhance cell growth, motility and chemoresistance	[58]
TFAM	2021	Liver cancer	Downregulation	Promote metastasis	[112]
TFAM	2021	Breast cancer	Upregulation	Promote breast cancer development and metastasis	[113]
TFAM	2020	Sarcoma	Downregulation	Inhibit cell proliferation	[114]

A great deal of big data analyses was conducted focusing on mitochondria-related biomarkers as therapeutic targets of cancer. In 72 non-small cell lung cancer (NSCLC) cases, overexpressed MFF formed complex with the key regulator of mitochondrial OMM permeability VDAC1 to regulate cell apoptosis [49]. Similar phenomenon was also observed in 192 patients of prostate cancer [50]. Among prognosis study, a pan-cancer analysis demonstrated that low expressed MFN2 due to mitochondrial dysfunction was associated with poor prognosis of renal clear cell carcinoma [51]. In 321 breast cancer cases, patients with high expression of Pink1 had shorter overall survival [52]. Relevant mechanisms have been preliminarily explored. Zamberlan M et al. revealed the close association of up-regulated OPA1 with poor prognosis of breast cancer based on bioinformatic databases. In breast cancer cells with OPA1 knock-down, the expression levels of miR-148/152 family increased inhibiting tumor growth and invasion. Therefore, mitochondrial dysfunction caused by OPA1 might regulate invasion and metastasis of breast cancer via the miR-148/152 family [53]. A total of 522 differential genes were identified by Li et al. [54] through RNA sequencing in HCC cells with OPA1 knock-down and 33 of them had most significant changes in metabolic pathways, suggesting that mitochondrial dysfunction resulted from aberrant fusion might promote HCC cell proliferation by cellular metabolism. Some lncRNAs were found to form complex with NRF1 to activate mitochondrial biogenesis in HCC [55] and colorectal cancer patients [56]. NRF1 was also found to activate E2F1 as a transcription factor to promote HCC proliferation by a ChIP-seq analysis for NRF1 target genes [57]. Similarly, the knock-down of TFAM could promote cancer progression according to bioinformatic databases of 18 head and neck cancer cases [58] as well as ovarian cancer [59]. All the big data analyses showed that mitochondrial dysfunction exerted roles in various cancer, which provided statistical clues for cancer therapy to some degree.

## Disorder in mitochondrial dynamics and gastrointestinal cancer

**Fig. 2:**
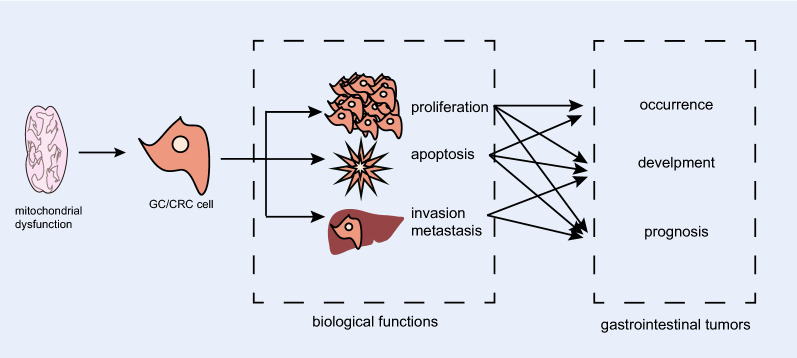
Disorder in mitochondrial dynamics and gastrointestinal cancer. Disrupted mitochondrial dynamics promotes the cancerization of gastrointestinal cellular biological behaviors, thus participating in the occurrence, development and prognosis of gastrointestinal tumor

Mitochondrial dysfunction due to disrupted dynamics has been referred in many tumors, while its role in gastrointestinal tumor is rarely studied. Exploration for the mechanism of mitochondrial dysfunction is a promising field in gastrointestinal tumor. Recent research found that mitochondrial dysfunction was involved in cellular biological behaviors and progression of gastrointestinal tumor (Fig. 2).

### Disorder in mitochondrial dynamics and cellular biological behaviors of gastrointestinal tumor

#### Disorder in mitochondrial dynamics and cell proliferation of gastrointestinal tumor

Mitochondrial over-fission enables tumor cells to proliferate in an unrestricted manner. Highly connected mitochondria could be observed in tumor cell cycle G1/S, which was thought to ensure a sufficient supply of ATP for tumor cell proliferation [115]. As a potential anticancer agent, PSII was found to inhibit colony formation and cell cycle arrest of G1 phase in HCT116 cell line, which might be caused by DRP1 knockdown inhibiting mitochondrial fission [116]. Therefore, disrupted mitochondrial dynamics may participate in cell proliferation of gastrointestinal tumor.

#### Disorder in mitochondrial dynamics and cell apoptosis of gastrointestinal tumor

Cellular apoptosis caused by disrupted mitochondrial dynamics has been extensively investigated. Yao et al. [117] believed that mitochondrial fission could increase ROS, activate caspase-9, induce cell apoptosis and decrease the viability of gastric cancer cells. Similarly, Somnath Mazumder et al. [118] suggested that indomethacin, a non-steroidal anti-inflammatory drug, might induce cell apoptosis of stomach cancer and impair mitochondrial dynamics by activating DRP1. Additionally, miR-148a-3p was identified as a miRNA with tumor suppressing effect. It was shown to enhance cell apoptosis induced by cisplatin through aggravating mitochondrial fission in gastric cancer cells intending to get better therapeutic effect, which was a first-line drug for treating locally advanced or metastatic gastric cancer [119]. Consistent result was also presented in mitochondrial membrane protein 18 (MTP18) for increasing DRP1 accumulation and promoting cell apoptosis of gastric cancer [120]. Moreover, under the stimulation of oxidative stress, the phosphorylation of atypical ERK sites in MFN1 increased the permeability of mitochondrial membrane and the oligomerization of apoptosis-related factor BAK (BCL-2 family member), promoted the release of cytochrome c and then cell apoptosis [121]. Therefore, any alteration in fusion and fission could disturb mitochondrial dynamics inducing cell apoptosis.

#### Disorder in mitochondrial dynamics and cell invasion & metastasis of gastrointestinal tumor

Mounting evidence suggested that disrupted mitochondrial dynamics was also involved in cell invasion and migration of gastrointestinal tumor. Mitochondrial fragmentation caused by over-fission increased the number of malignant cells and promoted the invasion of tumor cells in breast cancer [122]. FIS1 overexpression might be strongly associated with metastasis [81]. EBV virus was reported to induce mitochondrial fission by increasing DRP1 to promote Notch pathway-mediated migration of gastric cancer cells [123]. Besides, DRP1 knockout or Mfn1/2 overexpression was shown to increase fusion/fission ratio and significantly reduce migratory and invasive potentials of cancer cells [124]. Mfn1/2 overexpression in gastric cancer cells decreased their ability to migrate and invade, induced cell apoptosis via the PI3K-Akt pathway and impelled cell cycle to stagnate in G0/G1 phase [125].

All above-mentioned findings suggested that the disorder in mitochondrial dynamics affected malignant cellular biological behaviors of gastrointestinal tumor, and the key molecules could be targeted for tumor therapy.

### Disorder in mitochondrial dynamics and the genesis, progression and prognosis of gastrointestinal tumor

#### Disorder in mitochondrial dynamics and the genesis of gastrointestinal tumor

Disrupted mitochondrial dynamics has been confirmed to participate in all aspects of tumorigenesis. It has considerable prospect in the early and non-invasive diagnosis of gastric cancer. Mfn2 expression in normal gastric mucosa was found to be low and negatively correlated with tumor size. Moreover, Mfn2 could inhibit cell proliferation, induce apoptosis and weaken the invasiveness of gastric cancer by arresting cell cycle. Therefore, aberrant Mfn2 was linked to gastric carcinogenesis [125]. Chen et al. reported that p65 with its target genes cyclin D1 and c-Myc were down-regulated by knocking down DRP1 in HCT116 cell line, indicating that mitochondrial fission might inhibit colorectal carcinogenesis by activating the NF-kB pathway [116]. The antiallergic drug azelastine inhibited the IQGAP1-ERK-DRP1 pathway by targeting ADP-ribosylation factor 1 (ARF1), suppressed mitochondrial fission and colon carcinogenesis [66]. The aberrant expression of characteristic proteins causes disrupted mitochondrial dynamics and cancer initiation with the potential to be applied to gastrointestinal tumor treatment.

#### Disorder in mitochondrial dynamics and the progression & prognosis of gastrointestinal tumor

DRP1 was shown to be up-regulated in gastric cancer patients with cachexia, suggesting that mitochondrial dysfunction might be involved in the progression of gastric cancer [126]. Recently, BRAF ^V600E^ was revealed to be a quite common mutation in colon cancer. And a higher DRP1 level was also presented in colon cancer cells with BRAF ^V600E^ than in BRAF ^WT^ cells. Therefore, DRP1 might promote the progression of colorectal cancer driven by BRAF ^V600E^ [68].

The poor prognosis of gastrointestinal tumor is closely associated with tumor cell invasion and migration. In advanced gastric cancer of infiltrative (Borrmann III) and diffuse infiltrative (Borrmann IV) types, FIS1 expression increases and promotes cancer metastasis, indicating that the poor prognosis of gastrointestinal tumor may be associated with mitochondrial over-fission [81]. OMA1 is the precursor of different isoforms of OPA1. It was found to be highly expressed in gastric cancer and associated with poor prognosis [127]. Decreased Mfn2 also manifested the association with an aggravation in the stage of gastric cancer and a poorer overall survival [122]. Therefore, disrupted mitochondrial dynamics influences the progression and prognosis of gastrointestinal tumor.

## Summary and future direction

As the core of cellular metabolism, mitochondria appear to be vital in physiological process, pathological process and disease progression. The disorder in mitochondrial dynamics may regulate the cellular biological behaviors and progression of gastrointestinal tumor [83, 128, 129]. The mechanism about mitochondrial dysfunction with gastrointestinal cancer, however, remain poorly understood with the necessity to be further studied especially for the specific oncogenic pathways that mitochondrial dynamics participates in. Additionally, the clinical analysis of big data is also lacking for the expression of key molecules in mitochondrial dynamics during gastrointestinal cancer. Although the association of mitochondrial dynamics has been suggested with chemotherapy sensitivity of gastrointestinal cancer [119], more investigations are needed to support its roles in other aspects of cancer therapy such as drug resistance. The novel research has promising values in the diagnosis, treatment and prognosis of gastrointestinal cancer that cannot be neglected.

## Data Availability

Not applicable.

## Abbreviations

MQC: Mitochondrial quality control
mtDNA: Mitochondrial DNA
GTPase: Guanosine triphosphatase
GTP: Guanosine triphosphate
ATP: Adenosine triphosphate
ROS: Reactive oxygen species
DRP1: Dynamin-related protein 1
OMM: Outer membranes
IMM: Inner membranes
Cytc: Cytochrome C
MFN1/2: Mitofusion 1/2
OPA1: Optic atrophy 1
ER: Endoplasmic reticulum
MFF: Mitochondrial fission factor
Fis 1: Mitochondrial fission protein1
MiD49: Mitochondrial dynamics protein of 49kD
MiD51: Mitochondrial dynamics protein of 51kD
MTP18: Mitochondrial membrane protein 18
PGC-1α: PPARγ co-activator-1alpha
NRF: Nuclear respiratory factor
TFAM: Mitochondrial transcription factor A
SIRT1: Sirtuin1
PINK1: PTEN-induced putative kinase protein1
PARK2: Cytosolic ubiquitin E3 ligase, Parkin
BAT: Brown adipose tissue
